# Aging and Cardiovascular Disease: Current Status and Challenges

**DOI:** 10.31083/j.rcm2304135

**Published:** 2022-04-08

**Authors:** Mengge Zhou, Guanqi Zhao, Yuhong Zeng, Jiming Zhu, Feng Cheng, Wannian Liang

**Affiliations:** ^1^Vanke School of Public Health, Tsinghua University, 10084 Beijing, China; ^2^School of Medicine, Tsinghua University, 10084 Beijing, China; ^3^Center for Coronary Artery Disease, Division of Cardiology, Beijing Anzhen Hospital, Capital Medical University, 100029 Beijing, China; ^4^Department of Epidemiology, College of Preventive Medicine, Army Medical University (Third Military Medical University), 400038 Chongqing, China

**Keywords:** aging, cardiovascular disease, epidemiology, multimorbidity, deprescribing

## Abstract

Cardiovascular disease (CVD) is the leading cause of death worldwide. Population aging is becoming the most important driver of the CVD epidemic. With the rapid increase in an aging population, the burden of CVD will continuously increase. Most old people also suffer multimorbidity, which is strongly associated with impaired quality of life, disability, dependence, and mortality. However, few reviews evaluated the CVD burden accompanied by population aging and the challenges of CVD care in elderly individuals with multimorbidity. This review identified and summarized the current status of the CVD epidemic associated with aging and highlighted the challenges and needs of CVD care for the elderly.

## 1. Introduction

With the significant improvements in public health, sanitation, vaccination, socioeconomic development, public education, and health care, the epidemiological transition in the 20th century was accompanied by decreasing deaths and disability from communicable diseases but a continuous increase in noncommunicable diseases (NCDs) [1]. Of the types of NCDs, cardiovascular disease (CVD) is the leading cause of mortality and morbidity worldwide and will become more serious in the foreseeable future because population aging is progressing more quickly compared to the past [2]. With the aging population, the number of elderly individuals who are predisposed to developing incident CVD will continuously increase. With the improvements in health care, the number of survivors with CVD will also significantly increase. Therefore, the CVD epidemic due to rapid aging will become an urgent public health issue and bring new challenges to global health.

However, few reviews evaluated the CVD burden accompanied by aging and the challenges of CVD care in the elderly. Therefore, this review identified and summarized the current status of the CVD epidemic associated with aging and highlighted the challenges and needs of CVD care for the elderly to provide information for future research needs, policy formulation, and resource allocation.

## 2. Prolonged Life Expectancy and Accelerated Aging of the World Population

Life expectancy has significantly increased in the past several decades worldwide. Data from the Global Burden of Disease showed that life expectancy at birth increased 8.1 years (12.4%) from 1990 to 2019 (65.4 years in 1990 to 73.5 years in 2019) [3]. Approximately 80% of countries or territories had life expectancies at birth longer than 65 years in 2019 [3]. With improvements in life expectancy at birth, the life expectancy of the elderly is improving more rapidly [4]. The global estimate is that a person 65 years old should have expected to live an additional 17 years in 2015–2020, and this number may rise to 19 years in 2045–2050 [4].

**Fig. 1.**
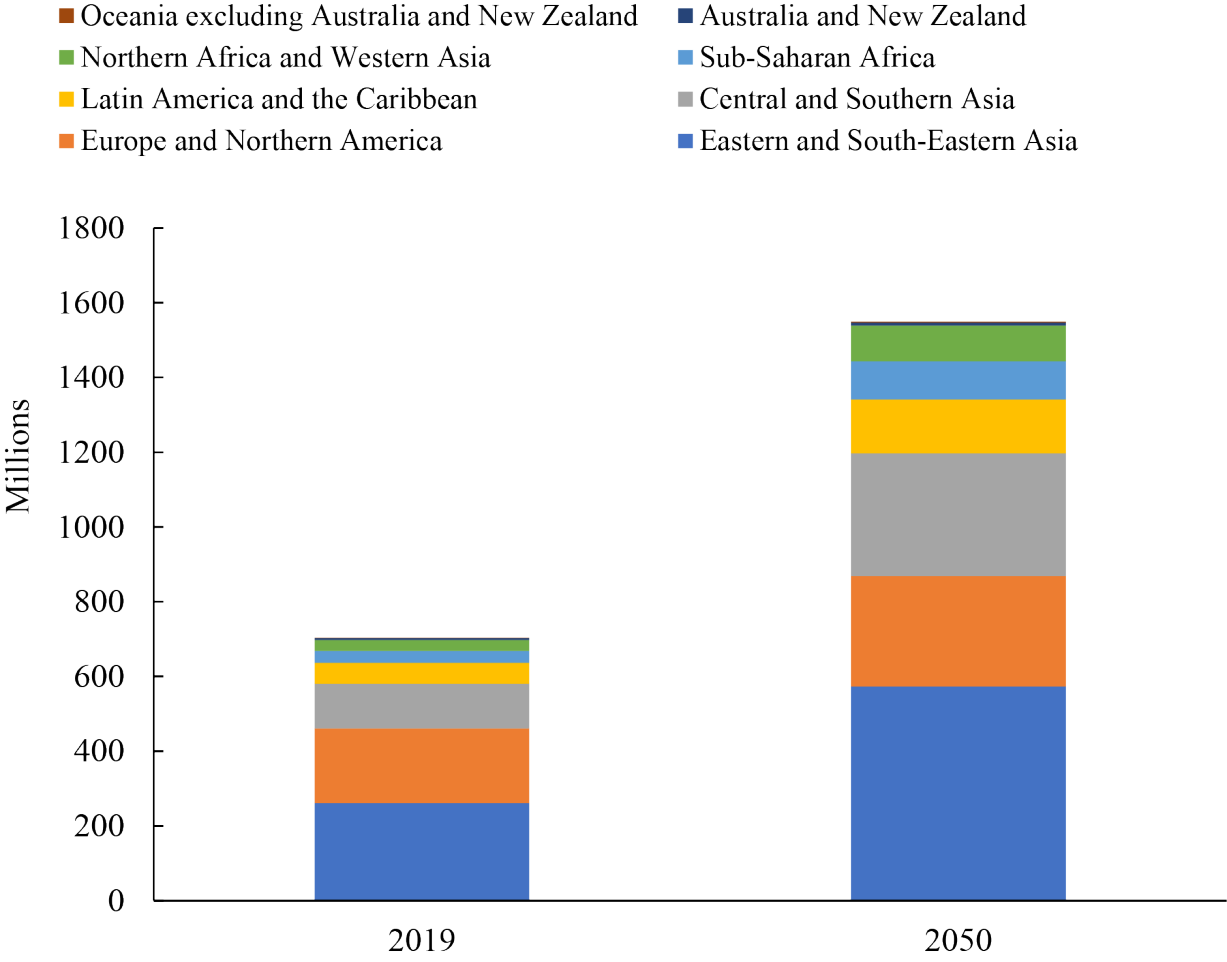
**Number of persons aged 65 years or over by region, 2019 and 2050 [4]**. The number of people aged 65 years or over would double from 700 million in 2019 to more than 1.5 billion in 2050 worldwide, and the largest number of older people were in Eastern and Southeastern Asia, with 261 million old people in 2019 and 573 million in 2050. By 2050, there will be more elderly people in Central and Southern Asia than in Europe and Northern America. Therefore, most developing countries will face a serious problem of rapid aging in the next 30 years.

World Population Prospects estimated greater than 700 million elderly people (age ≥65 years) in 2019 worldwide, and this number should be more than 1.5 billion by 2050, which represents nearly 15% of the world’s population [4]. Europe and North America are the most aging regions worldwide, with nearly 18% of the population being elderly in 2019, followed by Australia and New Zealand [4]. However, the largest number of older people were in Eastern and Southeastern Asia, with 261 million old people in 2019 [4] (Fig. 1, Ref. [4]).

However, a healthy, disease-free lifespan, i.e., healthspan, did not increase with lifespan [5]. An average of 16–20% of life is now spent in late-life chronic diseases, which are dominated by CVD, cancer, and neurodegenerative diseases [5]. Early estimates from the United States Vital Statistics demonstrated that eliminating CVD deaths would add 5.5 years to life expectancy [6]. Therefore, reducing CVD is very important to improve the quality of life of the elderly.

## 3. Heavy Burden of CVD in the Elderly

The total number of CVDs nearly doubled from 271 million in 1990 to 523 million in 2019, and the number of CVD deaths steadily increased from 12.1 million in 1990 to 18.6 million in 2019, which accounted for one-third of total deaths [3, 7]. Over 80% of all CVD deaths are attributable to two conditions, ischemic heart disease (IHD) and stroke, which are very typical age-related diseases [3].

**Fig. 2.**
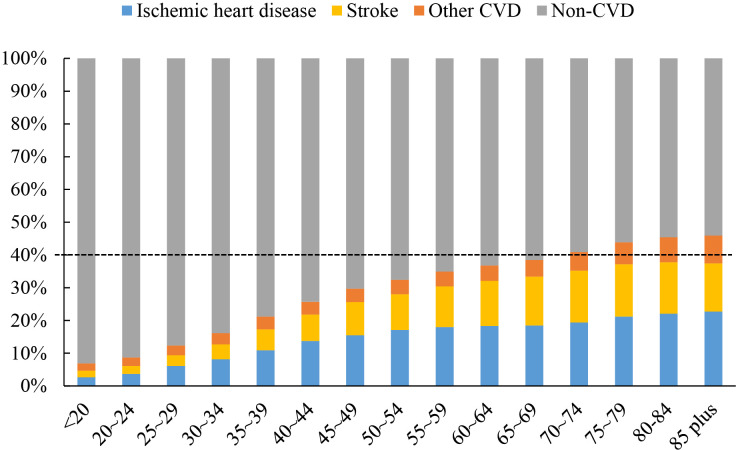
**Proportions of CVD in total deaths by age group [3]**. With increasing age, the proportion of CVD deaths, dominated by ischemic heart disease and stroke, to total deaths increased. Among people ≥70 years old, CVD accounts for greater than 40% of total deaths. CVD, cardiovascular disease.

**Fig. 3.**
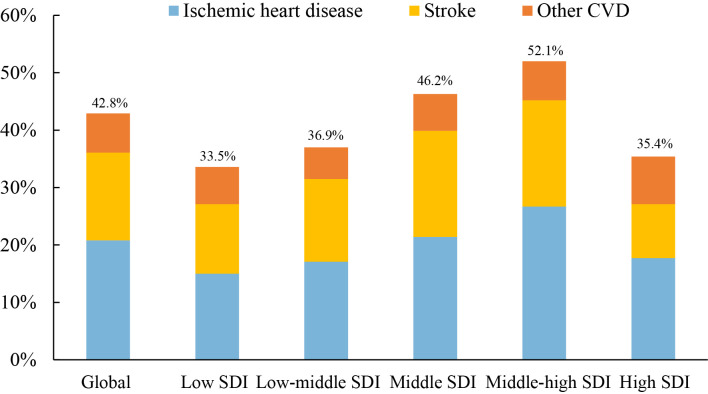
**Proportions of CVD in total deaths among people over 70 years by SDI group [3]**. Among people ≥70 years old, the highest proportion of deaths caused by CVD occurred in high-middle SDI regions, followed by middle SDI and low-middle SDI regions. It is foreseeable that the global deaths caused by CVD will continue to increase due to the continuously increasing mortality in middle- and low-SDI regions. CVD, cardiovascular disease; SDI, sociodemographic index.

With increasing age, the proportion of CVD deaths to total deaths increased (Fig. 2, Ref. [3]). Among people ≥70 years old, CVD accounts for greater than 40% of total deaths (Fig. 2), but large variations exist between regions with different sociodemographic indices (SDIs) [3] (Fig. 3, Ref. [3]). Contrary to our conservative perceptions that high-SDI regions have the highest CVD burden in the elderly, we observed that the highest proportion of deaths caused by CVD occurred in high-middle SDI regions, which is consistent with the three-stage theory of CVD epidemics proposed by Professor Dong Zhao [8] (Table 1, Ref. [8]). She summarized that high-income or developed countries featured the third stage of the CVD epidemic, which is characterized by a reduced proportion of CVD deaths and low premature CVD deaths but increases in cancer and dementia deaths. However, CVD mortality was quite high in the second stage of the CVD epidemic and accounted for a predominant proportion of the total deaths [8]. Therefore, high-SDI regions are in the third stage of the CVD epidemic, high-middle-SDI countries are in the second stage, and middle-SDI countries will quickly enter this stage. Therefore, the global deaths caused by CVD will continue to increase due to the continuously increasing mortality in middle- and low-SDI regions.

**Table 1.** **Three-stage theory of the CVD epidemic in different countries [8]**.

Stage	Characteristics of the CVD epidemic in different countries				Typical Asian countries
	CVD mortality	Spectrum of diseases (Proportion in total deaths)	﻿Proportion of premature CVD death in all CVD death	Life expectancy	
﻿Stage 1: Early stage of CVD epidemic	Low	CVD: approximately 20–30%	High, approximately 50%	Relatively short, approximately 65–70 years	﻿India, Nepal, and Pakistan
		CMNND: ﻿close to or greater than CVD			
		Cancer: much lower than CVD, approximately 10%			
		Dementia: very few			
﻿Stage 2: Stage of rapidly increasing CVD	High; rapidly increasing	CVD: quite high, even higher than 40%	Lower, 20–50%	Long, approximately 70–75 years	﻿Georgia,﻿ Armenica, Azerbaijan, Uzbekistan, Turkmenista,﻿Kazakhstan, China, Lebanon, Mongolia
		CMNND: ﻿fewer than 10%			
		Cancer: lower than CVD, ﻿but higher than stage 1			
		Dementia: low			
﻿Stage 3: Stage of decreasing CVD	Decreasing	CVD: high, but lower than stage 2, approximately 20–30%	Lowest, less than 20%	Longer, above 80 years	Japan, South Korea, ﻿Israel
		CMNND: ﻿fewer than 10%			
		Cancer: more dominant, more than 30%			
		Dementia: markedly increasing			

Abbreviations: CVD, cardiovascular disease; CMNND, communicable, maternal, neonatal, and nutritional diseases.

Although CVD is the main cause of death, case fatality decreased with improvements in medical treatment [9]. Therefore, the number of people who survive CVD is increasing [3]. The Global Burden of Disease estimated that there were 200 million people ≥70 years old suffering from CVD in 2019 [3]. This number will likely continue to increase with global aging. Recurrent CVD events are common in people who have already had a CVD, especially in the elderly [9]. Therefore, primary and secondary prevention should be addressed to reduce the burden of CVD.

## 4. Multimorbidity for Elderly Individuals with CVD

Multimorbidity is the coexistence of two or more chronic conditions, and it has become prevalent with an aging population and the decline in mortality [10]. The prevalence of multimorbidity is over 50% in the elderly and significantly increases with age [11]. Among people ≥80 years, multimorbidity is more common than any single disease, with over 80% of this population having two or more chronic conditions [12, 13, 14, 15].

Due to the high prevalence of CVD, CVD combined with other conditions has become the most common type of multimorbidity for the elderly. However, the combined conditions with CVD are complex, and the complexity increases with age. For example, the management of hypertension and IHD, two concordant conditions, is relatively easy in middle-aged and young-old populations. However, the management strategy becomes more complicated and controversial in the elderly. Different guidelines proposed different blood pressure targets due to different perspectives [16, 17, 18, 19, 20, 21, 22, 23, 24] (Table 2). The discordant conditions, which are less directly related to pathogenesis or treatment strategies [15], such as IHD and cancer, are often difficult or hopeless for specialists because current clinical guidelines and research primarily target single disease-specific care, and the evidence for co-treatment of discordant conditions is insufficient, especially for elderly individuals, who are often excluded or less represented in large-scale trials [10]. This situation should be urgently and extensively corrected because CVD rarely presents as an isolated disease in the elderly, and the number of elderly people with these comorbidities will explode.

**Table 2.** **Summary of the guidelines for the management of high blood pressure in adults and the elderly**.

Committee	Publication year	Population	Threshold to start therapy (mmHg)	Blood pressure target (mmHg)
International Society of Hypertension [16]	2020	<65 years	SBP >140/DBP >90	SBP <130 and DBP <80 if tolerated (However, SBP >120/DBP >70)
		≥65 years		SBP <140 and DBP <90 if tolerated but consider an individualized BP target in context of frailty, independence, and likely tolerability of treatment
Hypertension Canada [17]	2020	Low risk (no target organ damage or cardiovascular risk factors	SBP ≥160/DBP ≥100	SBP <140 and DBP <90
		High risk of cardiovascular disease	SBP ≥130	SBP <120
		Diabetes mellitus	SBP ≥130/DBP ≥80	SBP <130 and DBP <80
		All others	SBP ≥140/DBP ≥90	SBP <140 and DBP <90
Hypertension Branch of Chinese Geriatrics Society [18]	2019	≥65 years	SBP ≥140/DBP ≥90	SBP <140 and DBP <90
		≥80 years	SBP ≥150/DBP ≥90	SBP <150 and DBP <90
		≥65 years + frail	SBP ≥160/DBP ≥90	130 ≤SBP <150 and DBP <90
The Japanese Society of Hypertension [19]	2019	Adults <75 years	Lifestyle modifications should be attempted in all individuals with blood pressure ≥120/80 (high-normal blood pressure level or higher categories). In high-risk individuals with elevated blood pressure level and patients with hypertension (SBP ≥140/DBP ≥90), lifestyle modifications/non-pharmacologi-caltherapy should be performed actively, and antihypertensive treatment should be started as needed.	Office blood pressure
				SBP <140 and DBP <90
				Home blood pressure
				SBP <135 and DBP <85
		Adults ≥75 years		Office blood pressure
				SBP <130 and DBP <80
				Home blood pressure
				SBP <125 and DBP <75
NICE [20]	2019	<80 years	OBPM >140/90;	OBPM <140/90;
				ABPM/HBPM <135/85
		≥80 years	ABPM/HBPM mean ≥135/85 (measure orthostatic blood pressure in those ≥80 years or with symptoms of orthostatic hypotension)	OBPM <150/90
				ABPM/HBPM <145/85 (use clinical judgment for those with frailty or multimorbidity)
European Society of Cardiology [21]	2018	<65 years	OBPM ≥140/90;	SBP 120–129 for most and DBP <80
			ABPM ≥130/80;	
			HPBM ≥135/85	
		65–79 years	SBP ≥140/DBP ≥90	SBP 130–139 and DBP <80
		≥80 years	SBP ≥160/DBP ≥90	SBP 130–139 and DBP <80
American College of Cardiology [22]	2017	Adults with no history of CVD and with an estimated 10-year ASCVD risk <10%	SBP ≥140/DBP ≥90	SBP <130 and DBP <80
		Patients with clinical CVD or adults with an estimated 10-year ASCVD risk of 10% or higher	SBP ≥130/DBP ≥80	SBP <130 and DBP <80
		≥65 years	SBP ≥130/DBP ≥80	Ambulatory: Goal SBP <130
				high burden of comorbidity, limited life expectancy, clinical judgment, patient preference: assess risk/benefit
European Society of Hypertension [23]	2016	60∼79 years	SBP ≥140	SBP <130
		≥80 years	SBP ≥160	SBP 140–150
Joint National Committee 8 [24]	2014	All ages with DM and/or CKD	SBP >140/DBP >90	SBP <140 and DBP <90
		<60 years; no DM or CKD	SBP >140/DBP >90	SBP <140 and DBP <90
		≥60 years; no DM or CKD	SBP >150/DBP >90	SBP <150 and DBP <90

ABPM, ambulatory blood pressure monitoring; DBP, diastolic blood pressure; HBPM, home blood pressure monitoring; OBPM, Office blood pressure monitoring; SBP, systolic blood pressure.

### 4.1 Hypertension, Diabetes, Dyslipidemia and CVD

Hypertension, diabetes, and dyslipidemia are well-known risk factors and highly prevalent comorbid conditions of CVD [25, 26, 27], especially atherosclerotic cardiovascular disease (ASCVD), which is a combination of IHD and ischemic stroke.

**Fig. 4.**
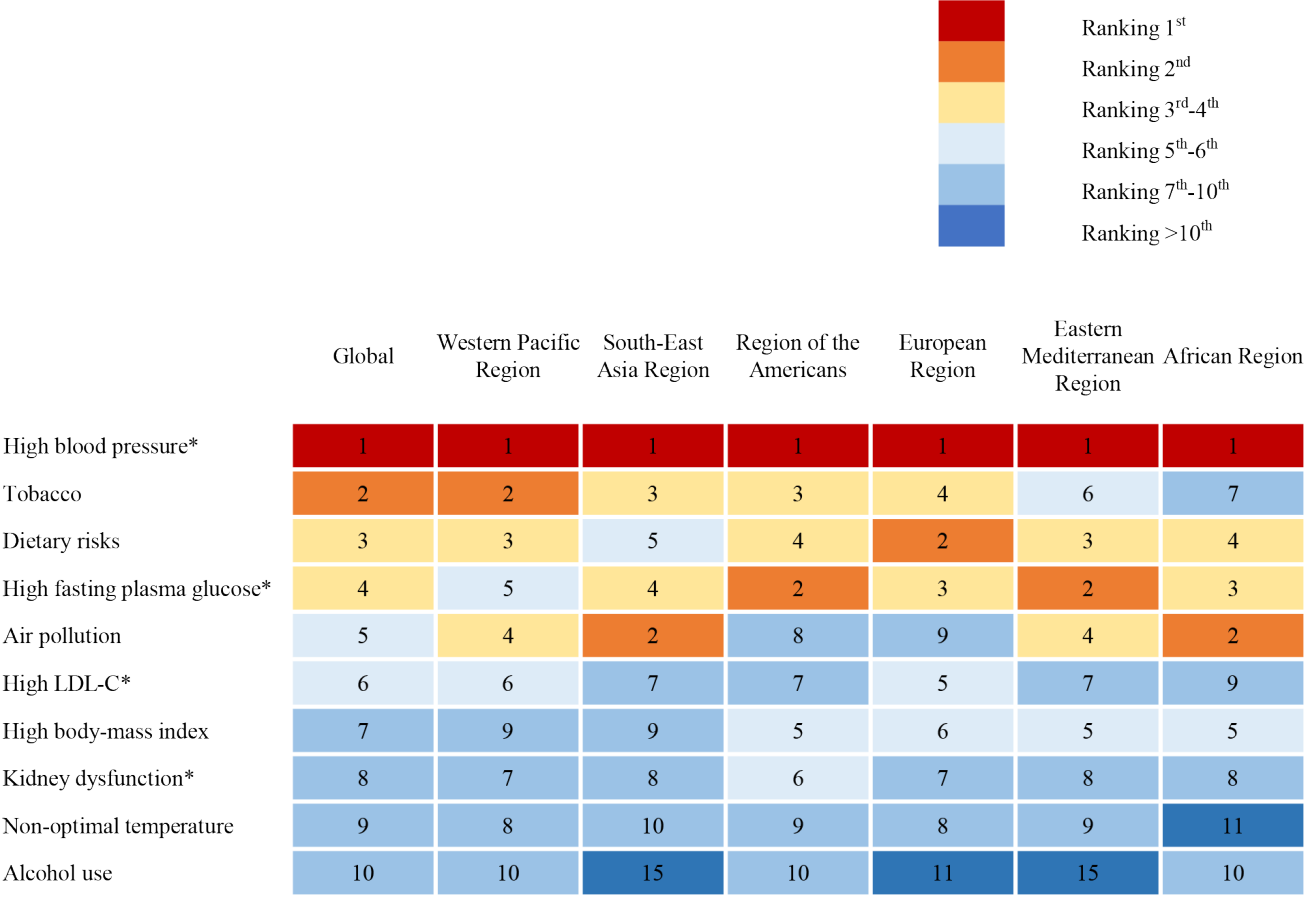
**Top-ranked risk factors contributing to death in people over 70 years by WHO region [3]**. The chart shows the top 10 ranked risk factors for death in people over 70 years of age. The number in the chart represents the ranking of risk factors and high blood pressure ranks first regardless of region. * Highly prevalent combined conditions of cardiovascular disease. WHO, World Health Organization; LDL-C, low-density lipoprotein cholesterol.

Among all of the risk factors for death in the elderly, high blood pressure ranks first regardless of region [3] (Fig. 4, Ref. [3]). Greater than two-thirds of elderly individuals with CVD likely have hypertension [28, 29]. However, a study in China found that only 13.0% of patients with hypertension and CVD had controlled hypertension [30]. Uncontrolled hypertension was associated with significantly increased risks for CVD mortality in 60- to 69-year-olds (risk ratio [RR], 2.6; 95% confidence interval [CI], 2.4–2.9) and 70- to 79-year-olds (RR, 1.9; 95% CI, 1.8–2.0) [30]. The Hypertension in the Very Elderly Trial (HYVET) of antihypertensive therapy for people aged >80 years found that lowering blood pressure was associated with a 39% reduction in the rate of death from stroke (95% CI, 1% to 62%; *p *= 0.05), a 21% reduction in all-cause mortality (95% CI, 4% to 35%; *p *= 0.02), and a 64% reduction in heart failure (95% CI, 42% to 78%; *p <* 0.001) [31]. The Systolic Blood Pressure Intervention Trial (SPRINT) found that management of systolic blood pressure (SBP) to a target of <120 mmHg was associated with a 34% reduction in the risk of cardiovascular events in people ≥75 years of age (hazard ratio [HR], 0.66; 95% CI, 0.51–0.85) and a 33% lower risk of all-cause mortality (HR, 0.67; 95% CI, 0.49–0.91) [32]. However, the results of these clinical trials are not easily generalizable to all patients with CVD, especially patients with heart failure or stroke, who are often excluded from clinical trials [31, 32]. Observational studies found that late-life blood pressure was decreased in the elderly, which was associated with excess mortality [33, 34]. Therefore, the goal of blood pressure for the elderly in different conditions is not clear and needs further study. For elderly individuals receiving antihypertensive treatment, home ambulatory blood pressure monitoring is recommended [35, 36, 37, 38].

Clinical guidelines classified patients with CVD and diabetes into extreme-risk groups [39]. At least one-third of patients with CVD have diabetes [40, 41]. With the rapid increase in the prevalence of diabetes in the general population, the proportion of diabetes in patients with CVD will likely continue to increase. A meta-analysis reported that diabetes was associated with a 1.7-fold higher risk of early mortality in patients with myocardial infarction/acute coronary syndrome (ACS), and the relative risk of early death associated with diabetes did not change over time based on the 86 studies published from 1970 to 2011 [42]. Zhou *et al*. [41] also found that diabetes was associated with a two-fold higher risk of in-hospital all-cause death and a 1.5-fold higher risk of major adverse cardiovascular events (odds ratio [OR], 1.54; 95% CI, 1.39–1.72) and a 2-fold risk of all-cause death (OR, 2.04; 95% CI, 1.78–2.33) in 2018. These findings suggest that advancements in the management of CVD patients during the last decades did not lead to a reduction in diabetes-induced risk. The use of SGLT-2 inhibitors or GLP-1 receptor agonists in patients with CVD and diabetes in clinical practice [43, 44, 45] are expected to reduce diabetes-induced risk.

Patients with a history of ASCVD are defined as a very-high-risk population [46], and an LDL-C goal of <1.4 mmol/L (55 mg/dL) or an LDL-C reduction of ≥50% from baseline are recommended [47]. However, a less stringent LDL-C goal of <1.8 mmol/L (70 mg/dL) was recommended for elderly individuals (≥75 years) with ASCVD. However, a national-representative study in China found that only 24.7% of hospitalized ACS patients ≥75 years with a history of ASCVD had LDL-C <1.8 mmol/L at admission [48]. Therefore, greater than three-fourths of older patients with ASCVD did not reach the LDL-C target level when they had recurrent events. The Cholesterol Treatment Trialists’ Collaboration performed a meta-analysis of individual participant data from 28 randomized controlled trials to evaluate the efficacy and safety of statin therapy in older people in 2019. It showed that statin therapy produced a 13% (RR, 0.87; 95% CI, 0.77–0.99) reduction in the risk of major vascular events and an 18% (RR, 0.82; 95% CI, 0.70–0.96) reduction in the risk of major coronary events with each 1.0 mmol/L reduction in LDL-C in patients ≥75 years old, which confirms that older patients receive a cardiovascular benefit from statin therapy [49].

Although multiple guidelines were issued and updated for the management of hypertension, diabetes, and dyslipidemia in adults and emphasize that the treatment goals of the elderly should not be too strict [21, 22, 47, 50, 51, 52, 53, 54], treatment strategies for elderly patients with CVD are far more complicated. Clinicians should provide personalized guidance based on the elderly’s overall health status and weigh the expected timing of benefits against life expectancy [47, 55, 56] based on the currently limited research evidence and clinical experience.

### 4.2 Kidney Disease and CVD

The glomerular filtration rate (GFR) steadily declines with normal aging, but this process may be influenced by superimposed diseases, such as hypertension, diabetes and CVD [57]. A recent study reported that 57% of patients with ACS aged ≥75 years undergoing successful percutaneous coronary intervention (PCI) had an eGFR of 30–59 mL/min/1.73 $m^{2}$, and 11% had an eGFR <30 mL/min/1.73 $m^{2}$. Therefore, two-thirds of elderly patients ≥75 years have moderate to severe renal dysfunction [58]. Compared to the patients with eGFR >60 mL/min/1.73 $m^{2}$, elderly ACS patients with an eGFR 30–59 mL/min/1.73 $m^{2}$ had a 1.65-fold (95% CI, 1.01–2.70) risk of all-cause death and a 1.77-fold (95% CI, 0.95–3.30) risk of cardiovascular death. For patients with an eGFR <30 mL/min/1.73 $m^{2}$, the risks of all-cause death and cardiovascular death were as high as 2.86 (95% CI, 1.52–5.36) and 3.11 (95% CI 1.41-6.83), respectively [58]. This result indicated that the higher risk of death associated with renal dysfunction in the elderly did not change over time [58, 59, 60]. The mechanism of the adverse impact of renal dysfunction for patients with ACS is multifactorial. Another consideration is that ACS patients with chronic kidney disease (CKD) are less likely to receive evidence-based therapies [60, 61]. For example, a 2013 systematic review and meta-analysis of 31 studies found that statin therapy reduced the risk of major cardiovascular events (23% RR reduction, 95% CI, 16–30) in patients with CKD, including patients receiving dialysis [62]. The benefit of statins was even higher in elderly individuals (age ≥65 years) with CKD, with a 28% lower risk of major cardiovascular events. The Acute Coronary Syndrome Israel Survey (ACSIS) of 8945 consecutive ACS patients from 2006–2016 found that the discharge prescription of statins was negatively associated with eGFR. ACS patients with an eGFR >60 mL/min/1.73 $m^{2}$ had 95% statin prescription at discharge, patients with an eGFR 30–59 mL/min/1.73 $m^{2}$ had 90% statin prescription, and patients with an eGFR <30 mL/min/1.73 $m^{2}$ only had 78% statin prescription (*p <* 0.001 for trend) [63]. Therefore, implementing programs to improve the quality of care for elderly individuals with CKD is essential.

### 4.3 Geriatric Syndromes and CVD

With improvements in longevity, geriatric syndromes, generally including frailty, sarcopenia, cognitive impairments, depression, urinary incontinence, vertigo, and falls, have attracted increased attention in recent years [15].

Frailty is a biological syndrome that is characterized by hypofunction of multiple physiological systems and vulnerability to stressors [64]. Five to 17% of older adults are affected by frailty [65]. Frailty and CVD are closely related [66, 67]. Frailty leads to an increased incidence of CVD, and CVD accelerates frailty [67, 68]. Because the tools and cutoff values to define frailty vary between different studies, the prevalence of frailty ranges from 10% to 60% [68]. Many studies consistently demonstrated that frailty significantly increased the risk of CVD and mortality [67, 69]. The Outcomes of Sleep Disorders in Older Men (MrOS Sleep) study estimated that frailty was associated with 84% increased CVD mortality (hazard ratio [HR], 1.84; 95% CI, 1.35–2.51), when ignoring the competing risk [70].

With increasing lifespan, the population of elderly with cognitive impairment is also increasing. According to a meta-analysis, the median prevalence of cognitive impairment is as high as 20% in people ≥60 years [71]. However, CVD, such as coronary heart disease (CHD), stroke, atrial fibrillation, and heart failure, further worsens this burden as risk factors for cognitive impairment [72, 73, 74, 75, 76]. A meta-analysis reported that CHD and heart failure were associated with a 27% (pooled RR, 1.27; 95% CI, 1.07–1.50) and 60% increased risk of dementia (pooled RR, 1.60; 95% CI, 1.19–2.13), respectively [76]. Cognitive impairment affects the care of CVD because it is a risk factor for lack of medication adherence in older adults [77].

Urinary incontinence is also common in the elderly [78], and it is often exacerbated by heart failure and risk factors for CVD, such as obesity, hypertension, and diabetes [79, 80, 81]. Some commonly used cardiovascular drugs also increase the risk of urinary incontinence, such as loop diuretics, angiotensin-converting enzyme inhibitors (ACEIs), and alpha-blockers [81, 82, 83, 84]. Urinary incontinence seriously affects the quality of life and increases the risk of sleep disturbance, depression, and social isolation [81, 85].

Functional decline, sensory loss, frailty, sarcopenia, and falls are also common in elderly individuals and may affect cardiovascular care to varying degrees. Regardless of hospitalization or outpatient follow-up for CVD, it is an important time window to identify geriatric syndromes. Clinicians should provide professional evaluation and prevention advice [15, 66].

In summary, it is very important to identify multimorbidity for older adults with CVD and perform research. First, it is necessary to understand the burden of CVD combined with other diseases in the elderly in different countries and regions and identify the most common disease combinations and current treatment measures. Then, multidisciplinary expert discussions and targeted clinical trials should be initiated for the elderly to provide evidence for clinical practice. Multidimensional health outcomes, such as function, health status, and quality of life, in addition to death and disability, should be considered in these studies.

## 5. Treatment of CVD in the Elderly

### 5.1 Type of Medications

Because multimorbidity is common in the elderly, the treatment of these diseases relies heavily on medical therapy. The prevalence of polypharmacy, which is generally defined as the use of five or more medications [86], is high in the elderly. The “wave 6” of the Survey of Health, Aging, and Retirement in Europe (SHARE) database showed that the overall prevalence of polypharmacy was as high as 32.1% (95% CI, 31.5%–32.7%) in older community-dwelling older adults across 17 European countries plus Israel [87]. Polypharmacy was more prevalent in hospitalized patients with CVD. Using the treatment of ACS as an example, at least 5 core medications should be provided according to guideline recommendations, including antiplatelet drugs (e.g., aspirin and ${\mathrm{P2Y}}_{12}$ inhibitors), statins (or other lipid-lowering drugs), ACEIs/angiotensin receptor blockers (ARBs), and β-blockers [88, 89, 90, 91, 92, 93]. Proton pump inhibitors are often used to prevent bleeding. For patients with hypertension, additional antihypertensive drugs are needed, and antidiabetic drugs should also be provided for patients with diabetes [88, 89, 90, 91, 92, 93]. Therefore, polypharmacy is inevitable for patients with ACS. However, the benefits are less certain when the drugs are used in combination because few clinical trials evaluated the drug–drug interaction (DDI) of the combined use of these drugs. Emerging evidence links cholesterol metabolism with platelet responsiveness [94], but whether the combined use of intensive lipid-lowering therapy and loading antiplatelet therapy would increase the risk of bleeding, especially in elderly individuals who are at high risk of bleeding, needs further evaluation. It is difficult to perform randomized controlled trials for this type of research because of feasibility and ethical considerations. With continuous improvements in the electronic medical record system and the interconnection of big data, it is more practical to perform real-world research. Once the DDI is discovered and verified from these observational studies, the mechanism underlying the DDI may be investigated. Some drug combinations should be used with caution or avoided in the elderly.

**Fig. 5.**
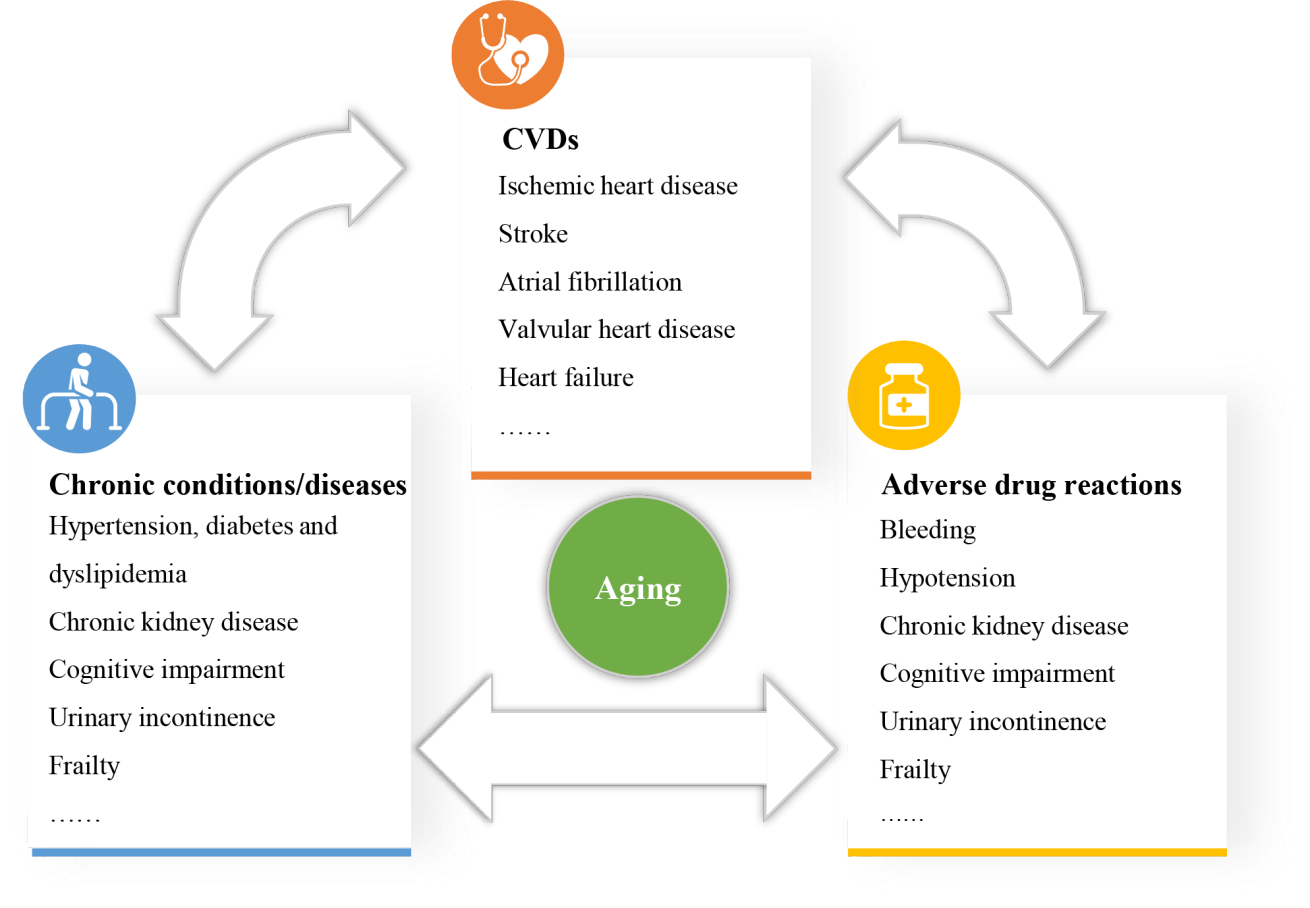
**The complex relationship between CVD, chronic conditions/diseases and adverse drug reactions**. Chronic conditions/diseases influence the occurrence and progression of CVDs. Meanwhile, CVDs can affect the management of chronic conditions/diseases. Multimorbidity results in polypharmacy, and polypharmacy is bound to increase adverse drug reactions. Adverse drug reactions will affect the treatment of CVD and other diseases, and finally, affect the prognosis of patients. CVD, cardiovascular disease.

In addition to the DDIs of different drugs, adverse drug reactions (ADRs), which is a more inclusive term, are more common in older patients [95]. ADRs occur in up to one-third of older outpatients and two-fifths of older hospitalized patients and account for one-tenth of all emergency department visits [96]. Patients using five or more drugs have an approximately 88% risk of ADRs, including an increased risk of malnutrition, renal insufficiency, metabolic disorders, bleeding, geriatric syndromes, and further decreased quality of life [97]. Therefore, the relationship between CVD, chronic conditions/diseases and ADRs is complex and interdependent (Fig. 5).

One study found that approximately two-fifths of the patients were taking one or more drugs that were deemed unnecessary [98]. With an evolutionary shift toward a “less-is-more” attitude for medication use, clinicians should comprehensively understand the ADRs and DDIs of polypharmacy, reduce unnecessary medications and develop an individualized medication plan for their elderly patients.

### 5.2 Dose of Medications

In addition to the compatibility of different types of drugs, the doses of drugs for the elderly are also worthy of attention [99]. The current guidelines recommend providing dual loading doses of aspirin and a ${\mathrm{P2Y}}_{12}$ receptor inhibitor to patients as early as possible or at the time of PCI, regardless of age [90, 91, 92, 93]. However, Zhao *et al*. [100] found that a dual loading dose of antiplatelet therapy was associated with an increased risk of major bleeding (HR, 2.34; 95% CI, 1.75–3.13) but not with a decreased risk of major adverse cardiovascular events (HR, 1.66; 95% CI, 1.13–2.44) compared to dual non-loading antiplatelet therapy in patients ≥75 years with ACS undergoing PCI, which supports the therapeutic heterogeneity between different ages.

Aging always results in a series of physiological and pathological changes, which narrow the therapeutic ranges of drugs and increase the risk of side effects. Therefore, the dose of different drugs should be separately evaluated and prescribed for the elderly.

### 5.3 Treatment Duration

One of the most concerning problems of patients with CVD is whether they need medications for life. Many current clinical medications for CVD do not have a time limitation and are routinely administered over many years, such as aspirin, statins, ACEIs/ARBs, and β-blockers [88, 89, 90, 91, 92, 93]. However, few studies evaluated the long-term efficacy and safety of these frequently administered medications. Rossello *et al*. [101] found that the average duration of follow-up in 30 secondary prevention trials examining the four core cardiovascular medications was approximately 3 years. Therefore, the long-term benefits and risks of many cardiovascular medications are not known, especially in older adults with multimorbidity. Fortunately, the issue of treatment duration has attracted more attention in recent years, especially for dual antiplatelet therapy (DAPT). ACC/AHA updated a 2016 guideline focused on the duration of dual antiplatelet therapy in patients with CHD [102]. The “DAPT score”, derived from the Dual Antiplatelet Therapy study, was recommended for deciding whether to continue DAPT in patients with coronary stent implantation [103]. Older age contributes to a low DAPT score, which suggests that this population is less favorable for prolonged treatment. A recently published randomized trial evaluated the appropriate duration of DAPT in patients at high risk of bleeding (age ≥75 years applied the criteria of high risk) after the implantation of a stent, and it found that one month of DAPT was not inferior to the continuation of therapy for at least 2 additional months based on the occurrence of net adverse clinical events (7.5% vs. 7.7%, *p <* 0.001 for noninferiority) and major adverse cardiac or cerebral events (6.1% vs. 5.9%, *p *= 0.001 for noninferiority). Abbreviated therapy also resulted in a lower incidence of major or clinically relevant nonmajor bleeding (6.5% vs. 9.4%, *p <* 0.001 for superiority) [104]. These results indicate that shortening the duration of DAPT in older adults should be beneficial. For patients with different health statuses and stages of life, the goals of treatment may be different. The needs of patients should be fully considered in the treatment process.

## 6. Deprescribing in Older Adults with CVD

Deprescribing is the process of medication withdrawal or dose reduction to improve the patient’s outcome/function, lessen the drug burden, and prevent drug-related adverse events [105]. However, barriers exist in deprescribing in the clinical practice of CVD [105]. First, the evidence for deprescribing is insufficient, although several randomized controlled trials found that deprescribing resulted in a potential reduction in mortality, falls, depression, and improvements in cognitive function and psychomotor function [105, 106, 107, 108]. Second, the attitudes of the patient’s families toward deprescribing may be negative because active and aggressive treatment has been deeply rooted in their hearts and they worry that deprescribing may raise patients’ concerns that physicians are “giving up” on them. Third, efficient communication lines between multidisciplinary teams are lacking. Clinicians from one specialty are particularly cautious and reluctant to remove medications prescribed by another specialty, which may have a risk of medical malpractice. Fourth, tools for deprescribing are not universally available for clinicians and patients [105, 109]. Several tools predominantly focused on the care of older adults [109], including the Assess, Review, Minimize, Optimize, Reassess (ARMOR) tool, the Good Palliative-Geriatric Practice (GPGP) algorithm, the American Geriatrics Society (AGS) Beers criteria, and Screening Tool of Older Persons’ Potentially Inappropriate Prescriptions (STOPP) criteria, should be referred for further tool development.

## 7. Conclusions

Population aging is becoming the most important driver of the CVD epidemic worldwide. With the rapid aging population, the burden of CVD will continuously increase, especially for middle- and low-SDI regions. Most elderly people also suffer from multimorbidity, which is strongly associated with impaired quality of life, disability, dependence, and mortality. The rigid application of clinical practice guidelines for single disorders may contribute to polypharmacy, adverse drug interactions, and unnecessary cost. Although many challenges in promoting deprescribing remain, we should prepare to better meet the treatment goals of the elderly. Good-quality integrated care and long-term care services for CVD and multimorbidity, should be provided for the elderly. Some countries developed national policies to support comprehensive assessments of the health and social care needs of older people, and we hope that more age-friendly cities, communities, and hospitals will be constructed.
